# Germany’s next shutdown—Possible scenarios and outcomes

**DOI:** 10.1111/irv.12827

**Published:** 2020-12-05

**Authors:** Maria Vittoria Barbarossa, Jan Fuhrmann

**Affiliations:** ^1^ Frankfurt Institute for Advanced Studies Ruth‐Moufang‐Straße 1 Frankfurt Germany; ^2^ Jülich Supercomputing Centre Forschungszentrum Jülich, Wilhelm‐Johnen‐Straße Jülich Germany

**Keywords:** COVID‐19, epidemic outbreak, mathematical modeling, non‐pharmaceutical interventions, wave breaker

## Abstract

With the rapid increase of reported COVID‐19 cases, German policymakers announced a 4‐week “shutdown light” starting on November 2, 2020. Applying mathematical models, possible scenarios for the evolution of the outbreak in Germany are simulated. The results indicate that independent of the effectiveness of the current restrictive measures they might not be sufficient to mitigate the outbreak. Repeated shutdown periods or permanently applied measures over the winter could be successful alternatives.

## 1 INTRODUCTION

The rapid increase of reported COVID‐19 cases in Germany has prompted policymakers to announce on October 28, 2020, a new period of stricter control measures starting on November 2, 2020.^1^ As of the first day of this “shutdown light,” Germany accounts for 545,027 reported SARS‐CoV‐2 infections and 10,530 reported deaths.^2^ The decision from Oct 28 stipulates softer measures than were applied in spring of 2020.^3^ In particular, schools maintain in person teaching, and all shops, not just grocery stores, keep their regular opening hours. The rising case and fatality numbers reported in Europe since early September ^4^ made people more aware of the risk of infection, but opposing opinions persevered and stimulated protests throughout the country. It is hard to quantify how the measures are going to change the course of the epidemic in Germany, as a match to the March/April shutdown is not exactly possible. Nevertheless, mathematical models allow simulating different scenarios of how the November shutdown might play out and what is likely to follow under various assumptions on the policies and public behavior adopted.

## 2 METHODS

**Figure 1:**
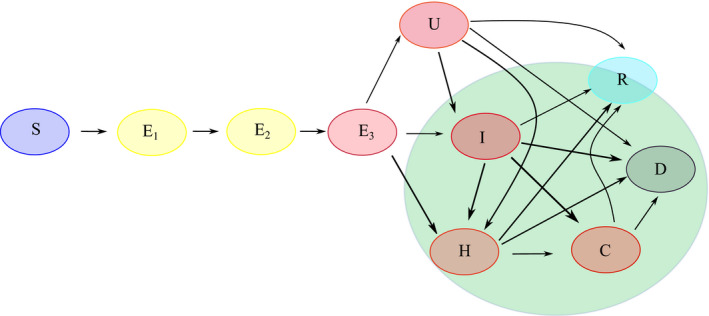
Model structure and transitions through the model compartments. Susceptible individuals (S) become exposed by effective contact with any infectious (red compartments). Exposed individuals progress through three stages, E_1_, E_2_, and E_3_, before illness onset. By testing and clinical diagnosis, both pre‐symptomatic (E_3_) and unknown (U) infectious individuals can be detected and thereby enter compartment I. Severe cases requiring hospitalization (H) or intensive care (C) are included. Infectious individuals are removed from the chain of transmission when they recover (R) or die (D) from the disease. Compartments covered by the green shadow are detected and reported in data. Unknown cases (U) lead to unknown recoveries

The results shown here are based on simulations of a mathematical compartmental model of SEIR (susceptible‐exposed‐infected‐recovered) type that in addition accounts for undetected and hospitalized cases and partitions the population into age classes. The compartments are summarized in Figure 1, and transitions between them are described by a system of ordinary differential equations comparable to what was proposed in.^5^ Susceptible individuals become exposed by effective contact with an infectious individual. Exposed individuals progress through three stages, *E_1_, E_2_,* and *E_3_,* before onset of possible symptoms. Individuals in stage *E_3_* are pre‐symptomatic but already infectious. By testing and clinical diagnosis, both pre‐symptomatic and unknown infectives can be detected. Detected or undetected diseased individuals can require hospitalization or even intensive care. By assumption, hospitalized and ICU patients are automatically reported as infectious, if not detected at an earlier stage. Hospitalized patients can reach a critical state and be relocated to ICU. All infected persons will eventually recover or might die from the disease. Importantly, individuals having recovered from undetected infection will never show up in official case counts. Individuals in any of the age groups considered here (juveniles: age 0‐14y; adult: 15‐59; senior: 60+) can be in any of the above states. Age classes evolve in parallel and are coupled to one another by contact rates among individuals. Demographics are neglected, meaning that besides fatalities caused by the disease, no individuals enter or leave the population. The model is calibrated on reported case counts,^10^ hospitalizations and ICU occupation as daily reported by the Robert Koch Institute,^2^ following methods previously adopted in.^5^

All the scenarios described below assume that (i) high incidence numbers lead to automatic contact reduction by making individuals on average more careful, (ii) effective contact rates are higher in winter than in spring/summer due to more frequent indoor activities,^6^ (iii) high prevalence leads to higher under‐reporting due to limited test and contact tracing capabilities, and that (iv) immunity acquired by having contracted and recovered from the disease does not wane as evidence on loss of immunity is yet debated.^7^, ^8^

**Figure 2:**
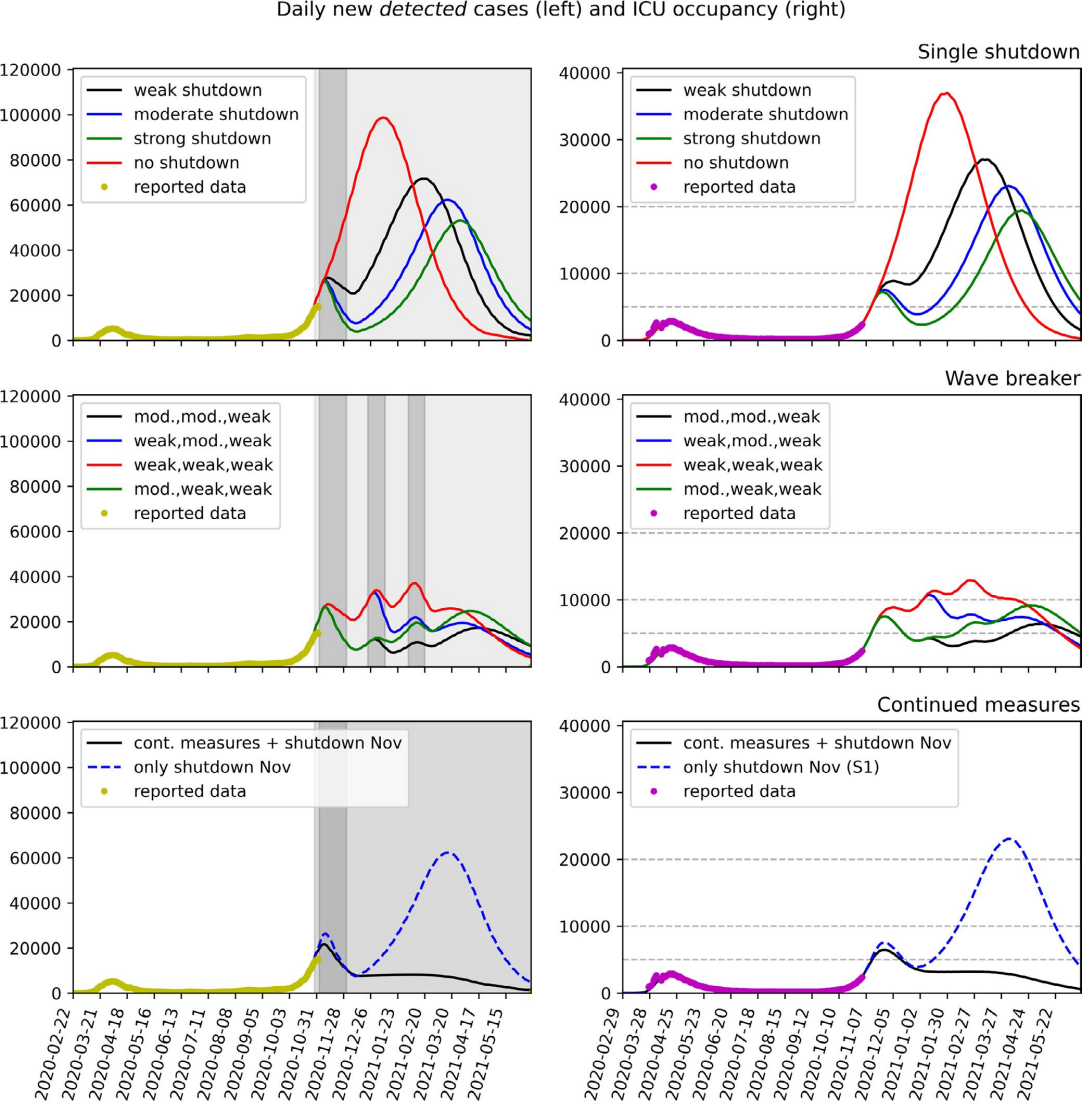
Scenario comparison. Left column: Model fit to RKI reported data (7 day moving average, yellow dots,^10^) up to October 27, 2020, and predictions for daily new reported cases. Right column: Solid lines show the model fit to reported data (purple dots,^11^) and predicted cases requiring intensive care; dashed gray lines show hypothetical critical thresholds of 5000, 10 000, and 20 000 occupied ICU beds (the latter taking into account an emergency buffer of approximately 10 000 beds), respectively. Top row: Scenario 1 (single shutdown). Starting on November 2, 2020, one single four weeks long shutdown period enhances earlier contact reduction measures. Possible levels of impact of the November measures are projected (red: no further measures—hypothetical; black: weak effect; blue: moderate; green: strong). Second row: Scenario 2 (wave breaker): Variations of the November shutdown are repeated during the Christmas (December 23 to January 11) and the carnival (February 1 to 21) period. Possible levels of impact of the measures on the daily reported new cases are projected. Third row: Scenario 3 (continuous intervention): Moderate restrictions as partially started at the end of October 2020 are maintained until spring 2021, coupled with four weeks more restrictive period in November 2020 (black curve). The blue curve shows comparison with the single November shutdown as in scenario 1

In what follows possible scenarios for reducing infectious contacts are considered. The simulations shown in Figure 2 report both the number of detected infections and the number of cases requiring intensive care, both being quantities of interest in the control of the pandemic. It should be emphasized that none of the scenarios simulates a shutdown similar to the one in spring 2020. The term “shutdown” is used here for lack of a better word to concisely describe the measures taken. Moreover, the term *severity of restrictions* can be more properly interpreted as *amount of reduction in effective contacts*.

- Scenario 1 (*single shutdown*): one shutdown period of four weeks (November 2 to December 1, 2020) is added to the earlier contact reduction measures (wearing masks, keeping 1.5 meter distance from others, washing hands, locally applied restrictions on opening hours for bars and restaurants). The impact of the planned measures being hard to estimate, we project in Figure 2 (first row) three possible levels (weak, moderate, strong effect), and compare with a model that does not introduce any further restrictions.
- Scenario 2 (*wave breaker*): Scenario 1 is enriched with two further shutdowns during the Christmas holidays (December 23 to January 11, 2021) and the carnival period (February 1 to 21, 2021). We project in Figure 2 (second row) four possible impact levels (weak, moderate, strong), possibly combining intervention packages of different severity.
- Scenario 3 (*continuous intervention*): In this scenario, moderate restrictions having partially started at the end of October 2020 are maintained until spring 2021, coupled with four weeks of more intensive restrictions in November 2020 (black curve in Figure 2, third row).

## 3 RESULTS

Mathematical models similar to the ones used here were employed in the early phase of the pandemic to follow and predict the outcome of initial intervention measures. One of the most salient predictions of these simulations was the inevitability of a second wave of the epidemic if the measures imposed during the first shutdown period in Spring of 2020 were to be relaxed too much.^5^ This outcome can be witnessed not only in Germany, but in many countries all over Europe and worldwide where strict interventions have been lifted stepwise during the summer. Now, a similar prediction concerning a third wave can be deduced from the simulations presented here.

As shown in scenario 1, no matter how effective the shutdown in November is in reducing contact rates and hence the incidence of new infections, returning to contact rates close to those prevalent in late summer will most likely lead to a third wave of rising case numbers that, if left unchecked, will lead to an even higher demand on the health care system as observed in either April or November of 2020.

Scenario 2 combines the November shutdown with additional shutdowns over Christmas and during the Carnival period, either predetermined at some time in fall or as spontaneous reaction to newly rising case numbers predicted for scenario 1. This alternation of periods with moderate restrictions and stricter contact reduction phases (shutdowns) allows to keep both the incidence and, probably more importantly, the required number of ICU beds under control, assuming the contact reduction during the shutdown periods is sufficiently strong.

Simulations of Scenario 3 indicate that continued moderate interventions over the whole winter might be a successful strategy to keep the number of new reported cases under control. Coupling these measures with a short, more restrictive period like the shutdown in November could allow to significantly dampen the current second wave for a few months from now.

Instead of planned wave breaker interventions, fixed for established periods of the year, one might also think of applying and relaxing intervention measures based on the reported case incidence. We have tested (simulations not shown here) also such an intervention strategy with triggered measures, assuming, for example, that an incidence of 50 cases per 7 days and 100,000 inhabitants triggers severe restrictions leading to strongly reduced contact rates, whereas lifting of these restrictions is triggered by the incidence dropping to 8 cases per 7 days and 100,000 inhabitants. One obvious assumption to include in the model is that control measures cannot be put in place instantaneously, but require a few days to be effectively introduced or relaxed. This leads to dynamics similar to that in scenario 2, but with shutdown periods not occurring at predefined intervals. This holds true even when the threshold values are modified to take into account rising detection ratios due to, for example, improved contact tracing or increasing testing capacity.

## 4 DISCUSSION

We conclude that the 4‐week soft to moderate shutdown started on November 2 cannot on its own be expected to prevent a third, possibly even stronger wave of COVID‐19. Repeated shutdown periods (with moderate to severe restrictions) could allow basic activities to be maintained, while keeping the COVID‐19 waves under control. Maintaining the measures already partially introduced in October until spring of 2021 together with some of the measures announced on Oct 28, and possibly combined with short‐term stronger restrictions, seem to promise the containment of the epidemic in Germany through the winter. Come spring, shifting of social activities to outdoors settings, possibly accompanied by availability of save and effective vaccines, may help suppressing the long winter outbreak.

We note that the observations above and simulations shown in Figure 2 are based on mathematical models that, as a matter of principle, only describe a part of reality. In particular, reactions of the population to increasing case numbers (more cautious behavior, self‐limitation of physical contacts) on the one hand and less than perfect compliance with officially mandated restrictions on the other hand may lead to significant deviations from theory. It is mathematically not possible, based on reported cases, to quantify the effects of any single intervention measure, as there have only been varying combinations of measures in the short history of COVID‐19 control. Possibly the strongest limitation of mathematical modeling in the context of COVID‐19 forecasts is that these are based on official case counts only, hence solely on detected cases. The unknown detection ratio, which is most likely fluctuating in dependence on new cases and testing capabilities, might significantly affect the outcome of model simulations.^9^ In order to model the occupation rate of ICU beds, we assumed that the same standard for admission to and release from intensive care is uniformly applied over time. Changing this admission policy over time in reaction to higher demand may lead to lower ICU occupation than shown in the model. Increasing the availability of ICU beds by, for example, establishing temporary field hospitals might be a further mitigation strategy for coping with even higher ICU occupancy. If this strategy is considered, the timing of maximal ICU demand may be as relevant as the number of beds required when assessing different scenarios. Finally, the presented model results cannot—and do not intend to—make any statements about possible economic or social effects of contact restrictions.

## CONFLICT OF INTERESTS

The authors declare no conflict of interests.

## AUTHOR CONTRIBUTION

**Maria Vittoria Barbarossa:** Conceptualization (equal); Formal analysis (equal); Investigation (equal); Methodology (equal); Visualization (equal); Writing‐original draft (equal). **Jan Fuhrmann:** Conceptualization (equal); Data curation (lead); Formal analysis (equal); Investigation (equal); Methodology (equal); Software (lead); Visualization (equal); Writing‐original draft (equal).

## AUTHOR CONTRIBUTIONS

MVB JF: Conceptualization; methodology; writing. JF: Numerical simulations.
